# Practical Departments

**Published:** 1901-07-20

**Authors:** 


### PRACTICAL DEPARTMENTS.

### PAPYROLITH JOINTLESS FLOORING.

(Papyrolith—formerly Asbolith—Flooring Company, Limited, Dashwood House, Old Broad Street, London, E.C.)

What is Papyrolith? The name suggests a substance of the nature of paper, or papier maché, as Asbolith suggests something to do with asbestos. Asbolith and Papyrolith are in fact different names for the same thing, the latter having been substituted for the former. The proprietors of Papyrolith claim that it is a tough jointless flooring of an absolutely fireproof nature; that it is sound proof and impervious to wet, and a most valuable material for floors of hospitals, asylums, &c., where it is important to avoid joints or cracks in which dirt can accumulate, and pathogenic organisms secrete themselves; that it resembles somewhat the nature of wood, being warmer to the feet than artificial stone, cement or asphalte; that it is elastic, deadens sound, and has many of the advantages of wood without its disadvantages of inflammability and difficulty of cleaning. If Papyrolith were all this, an almost perfect floor-substance would have been produced; but unfortunately it has been found that such hopes were fallacious. As to its sound proof qualities, we have nothing to say; but we have unmistakable evidence of its absorbent properties, and this is in itself enough to condemn a flooring for hospital purposes.

In the clinical laboratory of one of the London hospitals, we saw the Papyrolith floor bespattered with stains which we were assured could not be removed, and our attention was drawn to the appearance of the surface, which showed "the direction of the boards beneath." Obviously an "elastic" substance which "attaches itself inseparably" to its foundation is bound to adapt itself to the uneven surface of the old boards beneath. In the same hospital there is an oblong block of this material let into the floor of the entrance-hall, where there is no doubt a great deal of traffic over it, and it has a cut-up, chipped, and altogether old and untidy appearance.

An article of the kind is sadly wanted, but we must have something better than this.

